# Exploring the drivers of inter- and intraspecific differences in prosociality in four parrot species

**DOI:** 10.1038/s41598-025-04115-z

**Published:** 2025-07-02

**Authors:** Désirée Brucks, Nick C. P. Dam, Anastasia Krasheninnikova, Bethan McGregor, Hari Tsivlin, Auguste M. P. von Bayern, Jorg J. M. Massen

**Affiliations:** 1 Max Planck Institute for Biological Intelligence, Eberhard-Gwinner-Straße, 82319 Seewiesen, Germany; 2 Loro Parque Fundación, Max-Planck Comparative Cognition Research Station, Puerto de la Cruz, Tenerife Spain; 3 Animal Husbandry and Ethology, Thaer-Institute of Agricultural and Horticultural Sciences, Humboldt Universität zu Berlin, Berlin, Germany; 4 Institute of Biology, Leiden University, Leiden, The Netherlands; 5 Animal Behavior & Cognition, Department of Biology, Utrecht University, Padualaan 8, Utrecht, 3584 CH The Netherlands; 6 Avifauna Bird Park, Alphen aan den Rijn, The Netherlands

**Keywords:** Avian cognition, Cooperation, Cooperative breeding hypothesis, Group service paradigm, Interdependency hypothesis, Self-domestication hypothesis, Animal behaviour, Behavioural ecology

## Abstract

Prosocial behaviours - behaviours that benefit others at low or no cost to the actor - have been associated with cooperative breeding, nesting ecology, and overall levels of dependency and social tolerance. To confirm whether these factors drive the evolution of prosociality, we need to generate more robust data using a comparative approach with a standardised procedure. Parrots present a compelling model group to test this further due to their large brains, and advanced cognitive abilities. Using the group service paradigm, in which individuals can provide food to group memebers, we tested four distantly related parrot species with different socio-ecological backgrounds. All species provided food to group members at moderate to high levels and some individuals of all species were able to discriminate between situations in which food could be provided to others or not. All parrots demonstrated dyadic preferences by providing more food to both affiliated and non-related partners, with sex-specific effects varying between species. Furthermore, males from species that engage in cooperative breeding and territorial nesting provided the most food to members of their group. This implies that cooperative breeding and a reliance on other group members may be driving factors in the evolution of prosocial behaviour in parrots.

**Supplementary Information:**

The online version contains supplementary material available at 10.1038/s41598-025-04115-z.

## Introduction

Intentional and voluntary benefitting other individuals is referred to as prosocial behaviour^1^. Helping unrelated individuals at a cost to the actor is at odds with the evolutionary arms race, in which all individuals try to achieve the best possible fitness outcome for themselves and their kin^2^. Despite this theoretical assumption, humans^3^ but also some non-human species tend to display prosocial concern for each other. Typically, prosociality is tested by giving an individual the opportunity to share food with others or providing a choice between a selfish and a prosocial option without incurring any costs (for reviews see^4,5^). Responses to such tests vary considerably between species (e.g^6–8^vs^9,10^). , and could not be explained by phylogenetic relationships. Accordingly, it has been suggested that socio-ecological factors affect the emergence of prosocial behaviour convergently^11^.

Various hypotheses have been put forward to account for inter-specific variation in prosociality. According to the self-domestication hypothesis, prosociality might be considered a by-product of selection for reduced aggression. Less aggressive species show enhanced social tolerance, which enables prosocial behaviour^12^. In line with this hypothesis, bonobos (*Pan paniscus*), which are considered to have undergone self-domestication^12^showed more prosocial behaviours^13^ than the less socially tolerant chimpanzees (*Pan troglodytes*)^14^yet several other studies show the opposite pattern^8,15–17^. Alternatively, it has been proposed that cooperative breeding might be an underlying factor mediating prosocial concern^18^ as allomaternal care involve many instances of proactive help in the form of food provisioning or other care-taking behaviours towards related young conspecifics. This hypothesis was supported by a large comparative study that tested 15 different primate species^11^but see^19^*).* Cooperative breeding also emerged as an explanatory factor in a comparative study on corvids^20^ ), an avian group. In addition, colonial nesting and thus reduced aggression and increased social tolerance towards familiar group members (cf. self-domestication hypothesis^12^) were likewise linked to prosociality in corvids, however, revealing opposite sex effects. Since also non-cooperatively breeding species with low social tolerance exhibit prosocial behaviours (i.e. Japanese macaques^21^), it has been suggested that interdependency (e.g., for coalition formation) in dyadic relationships should also be considered as a predictor for prosocial behaviour, in addition to group-level interdependency related to social tolerance or cooperative breeding. The interdependency hypothesis underlines the value of prosociality towards group members in general (e.g., when breeding, group cohesion or nesting opportunities are dependent on group members), and towards specific group members (e.g., when animals are dependent on specific others for alliances to overcome the Machiavellian challenges, like self-interest, competition and deception, in complex and hierarchical societies^21,22^).

To investigate the evolutionary drivers of prosociality, a systematic comparative approach is needed that allows for a valid comparison among species. The group service paradigm^23^ is a setup that can be easily adapted to different species. It was originally developed for primates but has been successfully adapted to birds^24,25^and this adaptation has been validated again in common marmosets^26^ (*Callithrix jacchus*). In the group service paradigm, one individual can provide food to other group members by pulling in a board^23^or landing on a perch^24^ attached to a seesaw. This results in a food reward becoming reachable at a second position on the apparatus, out of reach of the operator. Accordingly, an individual can provide food to others but cannot reach the food him/herself, since leaving the platform or releasing the handle will result in the reward becoming out of reach again. A standardised stepwise training procedure ensures that each individual has understood the task’s contingencies (i.e., landing results in food becoming available) prior to the test phase and likewise several control conditions are implemented (i.e., no food available and food not accessible) to tease apart different possible underlying motivations. Furthermore, the whole group rather than pre-selected dyads can be tested, thus, allowing for a more natural test environment, in which natural social interactions are possible.

Parrots, which are distantly related to the previously tested corvids but exhibit comparably larger relative brain sizes and comparable cognitive abilities^27^represent an interesting model group to further investigate the evolutionary drivers of prosociality. Like corvids, parrots exhibit very high neuron densities in their brains^28^ and pallial structures that are considered equivalent to the mammalian brain (i.e., neocortex^29^). Accordingly, parrots, like corvids, have evolved advanced cognitive abilities that could be essential for prosocial behaviour and a concern for the wellbeing of others (e.g., theory of mind; see^30^ for a review). Previous studies have documented prosocial behaviours in some parrot species; thus, suggesting that this trait has evolved in parrots. Cockatiels (*Nymphicus hollandicus*) have been observed to share food with multiple unrelated partners of their group^31^. African grey parrots (*Psittacus erithacus*) but not blue-headed macaws (*Primolius couloni*) helped others to obtain food by proactively providing them with tokens in a dyadic token exchange paradigm^32^. Similarly, Goffin cockatoos (*Cacatua goffiniana*) flexibly provided tools to partners that were necessary for retrieving a food reward in a dyadic tool transfer task^33^. And finally, in a prosocial choice task, in which parrots could choose between rewarding either just themselves or themselves and their partner, African greys^34^ (but see^35^) and Keas^36^ (*Nestor notabilis*) showed a preference for the prosocial option but apparently failed to grasp the task’s contingencies and chose the prosocial option also when this did not benefit the partner. The results of these first studies examining prosocial behaviour in different parrot species, however, as of yet preclude drawing conclusions about the socio-ecological factors that affect the occurrence of prosociality within parrots. Not only do the experimental paradigms that have been used differ in terms of task complexity, they also reveal inconsistent results^32,34,37^and additionally different training regimes, housing conditions, and individual experiences with humans hamper any direct comparison.

The current study aimed at collecting more robust data on the evolution of prosociality in parrots within and across taxa by applying a systematic comparative approach to parrots. We employed the group service paradigm, thus, the identical methods previously used on primates^11^ and corvids^20^ on four parrot species. We selected species from different regional distributions (i.e. Africa: African grey parrot (AGP); South America: blue-headed macaw (BHM); Oceania: eclectus (EC), *Eclectus roratus*; Australia: galah (GA), *Eolophus roseicapilla*), which are all distantly related, sharing their last common ancestor at around 60–85 million years ago^38,39^. Thus, we keep the influence of phylogeny to a minimum. Furthermore, the species selected varied in breeding behaviour (i.e. cooperative breeding vs. biparental and maternal care^40^) and nesting ecology (i.e. colonial vs. territorial nesting^41^) so as to investigate whether similar evolutionary forces act on prosociality in parrots compared to corvids^20^. Cooperative breeding is exceptionally rare amongst the 358 extant parrot species^40^; however, the eclectus parrot exhibits a cooperative breeding system, in which multiple males provide food to multiple single females that incubate the eggs^42,43^. African grey parrots^44–46^ and blue-headed macaws^47^ form long-term monogamous pair-bonds and only one male provides support during breeding. Galahs exhibit biparental incubation and males do not provide food to females during nesting^48^; furthermore, they nest in large colonies, in which multiple pairs share a nesting tree without overt aggression towards other pairs^49,50^. Territorial nesting (i.e., defending the nest against other conspecifics) has been described in African grey parrots^51^ and eclectus^43^. Concerning the blue-headed macaws, little information is available; however, only pairs have been observed around nests^47^suggesting that they can be considered a territorial nesting species (see Table 1 for an overview on social and breeding biology of the parrot species in the present study).

Based on the cooperative breeding hypothesis^18,52^we predict that cooperatively breeding eclectus parrots provide more food to group members (i.e. males that usually provide food to females) compared to the other three non-cooperatively breeding species. Furthermore, according to the self-domestication hypothesis^12^ reduced aggression (e.g., in a nesting context; increased social tolerance due to nesting in close proximity) and resulting enhanced social tolerance are linked to prosocial behaviour; consequently, we predict to observe more prosocial behaviours in the colonial nesting galah compared to the territorial nesting species. According to the more overarching interdependency hypothesis^21^ however, we predict that instead of species-level socio-ecological factors, individual relationships within the groups (i.e., affiliative relationships and kinship) are a better predictor for prosociality.

## Methods

### Subjects

We tested 39 captive bred birds of four parrot species in this study (AGP: *N* = 8 birds (6 F/2 M); BHM: *N* = 7 birds (4 F/3 M); EC: *N* = 5 birds (3 F/2 M); GA: *N* = 19 birds (10 F/9 M)). The AGP and BHM were tested at the Max Planck Comparative Cognition Research Station in the Loro Parque, Tenerife, Spain. The EC and GA were tested at Avifauna bird park in Alphen aan den Rijn, the Netherlands. Permission was obtained from Avifauna bird park and Loro Parque for the use of animals. For details on group composition (i.e. kinship, age) and housing of each species see SM. All parrots were group-housed in aviaries (AGP and BHM in same-species groups; EC and GA in one mixed-species group) with access to an outdoor aviary. Water was available *ad libitum* and the parrots were fed once or twice daily with a mix of vegetables, fruits, seeds, and pellets depending on the particular species’ feeding ecology.

### Apparatus

We used the same mechanism and apparatus as in Horn et al.^20^adjusted in size and weight for each of the four parrot species, which was attached to the mesh of the home aviary from the outside. The apparatus consisted of a wooden board that could move up and down using a seesaw mechanism (see Fig. S1). On the wooden board, two metal gutters were installed (provider position 0/receiver position 1) on which food items could be placed. Subjects were able to operate the seesaw by stepping or landing on the provider perch (position 0). A second perch (receiver perch – position 1) was directly fixed onto the wire mesh of the aviary (instead of fixed to the wooden board) and was thus unable to operate the seesaw mechanism. The apparatus was affixed to the aviaries at ~ 1.5 m height, thus, allowing the birds to reach the perches either by flying or climbing on the mesh. In the case of EC and GA, the perch could additionally be reached by walking on a metal bar that connected the mesh elements directly underneath the apparatus (see Video 1). In the resting position, the provider perch always pointed upwards, while the wooden board pointed downwards (see Fig. S1). By landing on the provider perch, the board moved downwards; consequently, food items rolled forward within reach of the birds. Once a parrot left from the provider perch, the board moved upwards again and the food item rolled to the back of the board and, thus, out of reach for the birds. If a subject landed on the provider perch, it could either provide food to itself (if food was placed in position 0) or to a group member (if food was placed at position 1). Parrots could land multiple times on the provider perch within one test trial since the seesaw could move back into the resting position. Thus, if a parrot left the provider perch before the food was taken by a group member, it became unavailable again. For the AGP and BHM, we used sunflower seeds, while for the EC and GA, we used blueberries as rewards.

### Training and testing procedure

The birds were trained and tested once per day at the same time of the day. Testing for the AGP took place Aug-Nov 2021, BHM Oct–Nov 2018, GA Mar–Jun 2021, and EC May–Jun 2021. A detailed description of the procedure is provided in the Supplementary Material. The training and test procedure followed the standard procedure of the previous studies^11,20,21,23,24,26^.

Following the habituation to the apparatus (phase I: see SM for details), a social tolerance test was conducted (phase II). In this test, which was spread over two sessions, single food items were placed on the fixed apparatus in receiver position 1 and it was noted which bird obtained the reward (see SM for details). Social tolerance was quantified by noting how evenly the food was distributed amongst the group members by calculating Pielou’s J’^53^ for each group (see Supplementary Material for details).

The subsequently calculated Pielou’s J, which represents the evenness of the food distribution- or rewards obtained per individual within each group in this test, was used as a measure of social tolerance within each group. Subsequently, the training started (phase III), in which the birds learned to operate the seesaw mechanism (i.e., movement of landing perch and location of food rewards; see SM).

Once all individuals (in the case of AGP, BHM, EC), and the majority of GA (*N* = 12), passed a training criterion (i.e. taking food repeatedly from the moving seesaw; see Table S1), the “group service phase” (phase IV) started (total sample size for phase IV & V was 32 birds: AGP: *N* = 8; BHM: *N* = 7; EC: *N* = 5; and GA: *N* = 12). In this phase, we assessed the parrots’ propensity to deliver food to group members. Two different conditions (test, empty control) were conducted on alternating days. In total, we conducted 5 sessions of each condition (thus 10 in total) and the number of test trials per session was dependent on the number of individuals within the groups tested (i.e., 5 trials/bird; e.g., for the EC this was 5 birds*5 = 25 test trials/session). In the test condition, food was placed at receiver position 1 and, therefore, food could be made available only to other birds by landing on the provider perch (position 0). To rule out that the presence of food triggered the landing behaviour, the empty control condition was conducted. In this control, no food was placed on the receiver position 1; however, the hand movement of baiting the apparatus was retained. Motivational trials were implemented in each session (both test and controls across all phases), initiating the session and subsequently each sixth trial of a session to ensure sufficient motivation throughout the study (e.g., for the EC this added 6 trials, leading to a total of 31 trials/session). During motivational trials, food was placed in provider position 0 and accordingly, the bird landing on the provider perch could get access to the food.

In the following “blocked phase” (phase V), access to food at the receiver position 1 was blocked with a transparent piece of plexiglass; accordingly, landing on the provider perch never provided food to a group member. This controlled for the possibility that the parrots’ landings on the provider perch were evoked by access to – or visibility of – the food. Again, as in the group service phase IV, a condition involving food being placed at the receiver position 1 was conducted (blocked control) and alternated with a condition without food but with the same hand movements (blocked-empty control), and each of these conditions was again tested in 5 sessions (i.e. 10 sessions in total).

And finally, after a break of 2–10 weeks, a “repeated phase” (phase VI) was conducted (except for the BHM due to time constraints), which repeated the test and the empty control condition of phase IV to control for order effects (i.e. decreasing motivation across sessions) and temporal stability of prosociality^19^. As in phase IV both conditions were tested on alternating days with two sessions per condition. We repeated these two conditions to account for order effects^11,20,21,23,24,26^. For further details on each phase and condition, please see the SM.

#### Behavioural observations

To assess dyadic preferences amongst the parrots, behavioural observations of the groups were conducted around the same time of testing (i.e., within 12 months prior and 4 months after finishing phase I - VI). Each species was observed for at least 12 h in total. Affiliative interactions (i.e. allofeeding, allopreening) and agonistic interactions (i.e. pecking, chasing) were noted *ad libitum.* Interactions between each possible dyad within a species were taken into account. Furthermore, all possible sex dyads were compared. Based on these data, we calculated affiliation scores per dyad by summing up the amount of affiliative interactions for each dyad and dividing this by the number of hours of observations we recorded for each dyad. We calculated an agonistic score using the same method. Since affiliation and agonistic score were correlated with another (see SM) and due to the fact that affiliation might be a better predictor for prosociality compared to undirected aggression rates^4^we used only the affiliation score for further analyses.

#### Coding and analyses

All phases were video-recorded and it was noted on a trial-by-trial basis, which birds landed on the apparatus at the provider position 0 and receiver position 1. Each trial lasted two minutes or until a food item was successfully delivered (in case of the test condition); consequently, individuals could land multiple times per trial at either provider or receiver position. In the group service phase, we additionally recorded the identity of the bird that successfully provided and received the food item. Furthermore we distinguished between proactive and reactive prosociality based on the presence of a recipient at the receiver position (as in^21,24^) and not based on begging behaviour of the recipient as in most primate studies^11,23^. If a bird was already present at receiver position 1 when another bird landed on the provider perch, it was coded as reactive, and if the receiver perch was empty when the provider bird landed, it was coded as proactive. Furthermore, we analysed if the bird that landed on the provider perch tried to access the food reward on the receiver side (i.e. reaching at least half the distance towards the receiver perch within 3 s after having landed on the provider perch; see Supplementary Material). The identity of landing individuals was scored live during the experiment by the experimenter and later confirmed by coding the video recordings. To assess interobserver reliability, 10% of videos were analysed by a second coder that was not involved in data collection (EC: all ICC ≥ 0.99, GA: all ICC ≥ 0.85, BHM: all ICC ≥ 0.98, AGP: all ICC ≥ 0.94).

The subsequent analyses were conducted in R^54^ (version 4.2.2). We only used data from the last two sessions per condition (i.e. Session 4 & 5 of each condition; i.e. test, empty- and blocked control) of the group service paradigm to ensure that all birds had sufficient experience to understand the contingencies (cf^11,20,21,23,24,26^). The data were analysed in five steps: (1) participation in motivational trials across sessions, (2) discrimination between test and control conditions on an individual level, (3) development of food provisions across sessions, (4) factors explaining species differences, and (5) dyadic variation in prosocial acts (see Table 1 for an overview and Supplementary Material for detailed descriptions).

For all models, we z-transformed continuous variables to a mean of zero and a standard deviation of one to facilitate model convergence and ease interpretability. All model assumptions were checked prior to fitting the model and are reported in the Supplementary Material. Furthermore, all full models were compared to null-models (only intercept) to assess the overall main effect of our predictors using Likelihood-Ratio tests (LRT).

**Table 1:** Overview and order of statistical analyses, including observations per data set.

#	Test	Data set	Response variable	Predictors	Random effect		Offset
Are all species motivated to participate?^A^							
1	GLMM (binomial)	Species-level *(AG: 120 obs*,* BHM: 105 obs*,* E: 75 obs*,* G: 180 obs)*	Landings on receiver perch in motivational trials/total trials (all sessions)	Sex + Condition * Session	(1+Conditon + Session|Bird ID)		–
Do parrots sustain food provisions across sessions?							
2	GLM (poisson)	Species-level *(AG: 5 obs*,* BHM: 5 obs*,* E: 5 obs*,* G: 5 obs)*	Food provided (all sessions)	Session	–	–	
Do they land according to task’s contingencies?^A^							
3	Chisq-Tests (individual level)	All birds tested in Phase IV & V (32 obs)	Individual number of landings on provider perch in Phase IV & V (S4 & S5)	–	–		–
What explains species differences?							
4	GLM (zero-inflated, negative binomial)	All birds tested in Phase IV & V (32 obs)	Sum landings on provider perch in test condition (Phase IV—S4 & S5)	Cooperation + Nesting + Social Tolerance	–		Trial number
To whom do birds provide food?							
5	GLMM (negative binomial)	Dyadic-level (*N* = 424 obs)	Food provided in S4&5	Species * (Kinship + Sex + Affiliation)	(1|Provider: Dyad) + (1 + Affiliation|Provider)		Trial number

^A^Results reported in Supplementary Material.

Lastly, two of the GA and two EC developed an alternative strategy (‘cheating’) to speed up the arrival of motivational trials during the control conditions; i.e., they repeatedly stepped on the provider perch at position 0 (at least 10 trials in a row) and earned the food reward during subsequent motivational trials (see Video 1). In the subsequent analyses, we have included these four birds, however, also report the results, when excluding them.

### Ethical note

All experimental procedures complied with the legal requirements of the Netherlands (Wet op de Dierproeven, WoD), German Animal Welfare Act of 25th May 1998, Section V, Article 7, and the Spanish Animal Welfare Act 32/2007 of 7th November 2007, Preliminary Title, Article 3 and the EU Directive 63/2010. Furthermore, we adhered to the Guidelines for the Treatment of Animals in Behavioural Research and Teaching^55^. The procedures were approved by the Avifauna Bird Park and the Loro Parque. As members of the European Association of Zoos and Aquaria (EAZA), Avifauna Bird Park and Loro Parque are bound by the legal and ethical animal welfare regulations. As the study was non-invasive, and did not cause any harm or discomfort in the animals, further ethical review and approval by any relevant body was not necessary.

## Results

### Species differences in landing patterns

Participation in the motivational trials was consistently high for all species (see Supplementary Material).

**Table 2:** Overview of socio-ecological variables and measurements obtained in the test per species.

Species	Coop. breeding	Nest type	Group Size	Pielou’s J‘	Phase IV provided food^a^	Phase VI provided food	Deliveries across sessions^b^	Av. Affiliation score^c^
*African grey*	No	Territorial	8	0.40	25.0%	82.5%	↓	0.96
*Blue-headed macaw*	No	Territorial	7	0.48	94.3%	NA	→	7.17
*Eclectus*	Yes	Territorial	5	0.80	98.0%	100%	→	5.94
*Galah*	No	Colonial	12	0.73	78.3%	46.7%	↑	0.21

^a^Percentage of successful food provisions in last two test sessions.

^b^Arrows depict trend of successful food deliveries across test sessions based on analysis of provided food items per session.

^c^Based on behavioural observations: sum of affiliative interactions per dyad/hrs of observations.

**Fig. 1:**
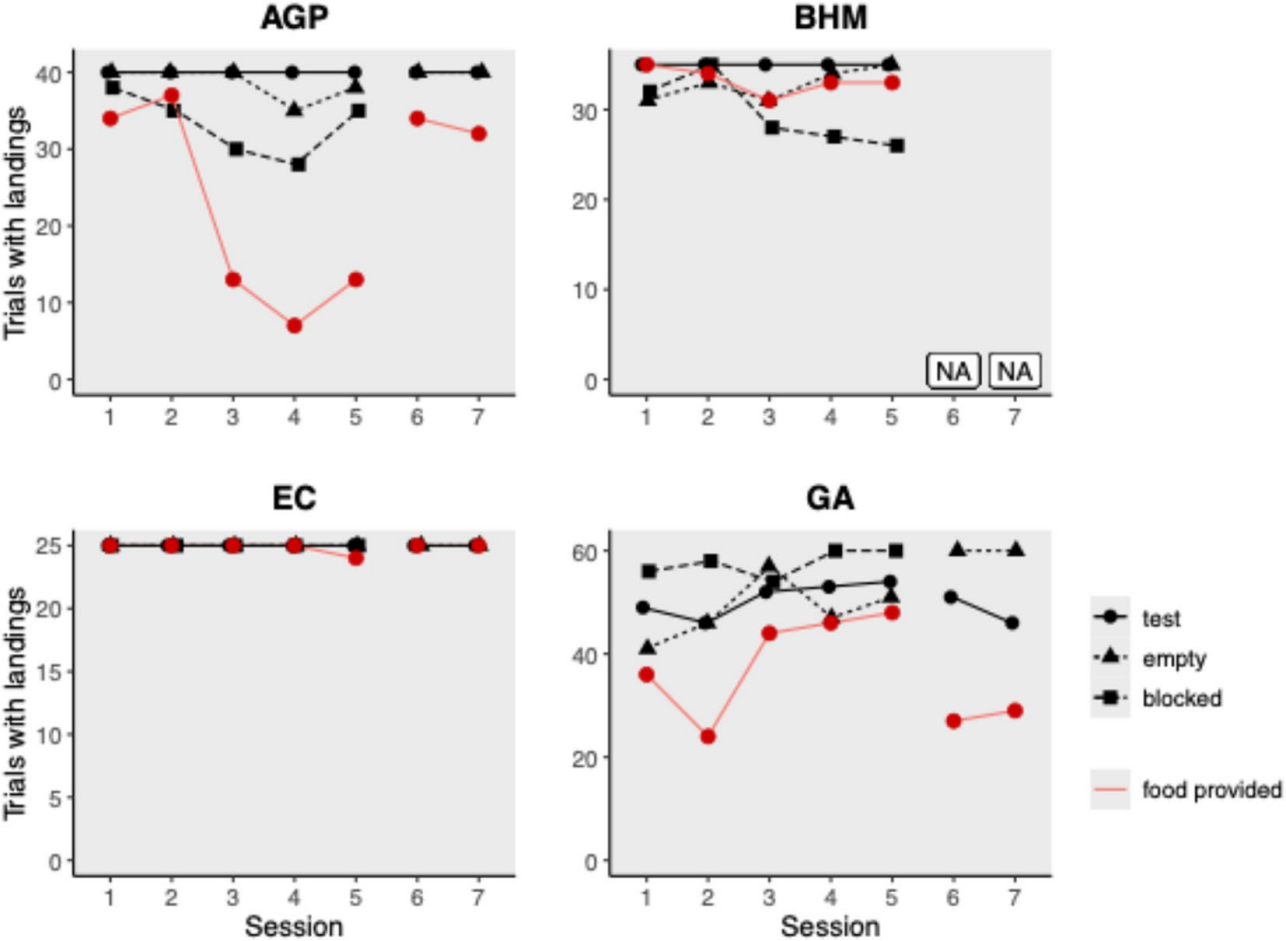
Number of trials with landings on provider perch across sessions in group service phase (IV) and blocked phase (V) plotted separately for each species (including all birds—see Fig. S2 for results excl. cheaters). The red line indicates the number of trials with successful food provisioning to another bird. *AGP* African grey parrots, *BHM* blue-headed macaws, *EC* eclectus, *GA* galah.

The participation rate differed between species with the EC being the most active and the AGP the least active species (mean number of landings on provider perch in test: AGP: 3.175 ± 5.992 landings per bird; BHM: 8.771 ± 9.178; EC: 10.480 ± 11.562; GA: 8.867 ± 11.620; see Fig. 1). While the AGP, BHM, and EC landed in every trial of the test conditions, successful food deliveries differed between species (see Tables 2 and 3). The AGP successfully delivered food to a receiver only in 60.7% of all trials with high provision rates in the beginning (1st session: 85%, 2nd session: 93%) and lower rates in the end (4th session: 17.5%, 5th session: 32.5%); thus revealing a clear decline in provisions across test sessions (GLM: -0.366 ± 0.075, z = -4.855, *p* < 0.001; see Fig. 1). The BHM provided food to others at a constant rate across sessions (94.9% of all trials, range: 88.6–100%; GLM: -0.015 ± 0.055, z = -0.274, *p* = 0.784; see Fig. 1). The EC provided food to others at a very high rate in almost every trial (99.2% of trials, range: 96–100%; 48.8% when excluding cheaters) and sustained this provisioning rate across sessions (GLM: -0.008 ± 0.064, z = -0.127, *p* = 0.899; excluding cheaters: GLM: 0.066 ± 0.091, z = 0.724, *p* = 0.469; see Fig. 1). The GA were the only species that did not land in every trial during the test condition (87.8% of trials; 64.7% when excluding cheaters). Successful food deliveries occurred in 60.5% of all trials (51.7% when excluding cheaters) with lower rates in the beginning (1st session: 60.0%, 2nd session: 40.0%) and higher rates towards the end (4th session: 76.7%, 5th session: 80.0%); thus, the GA increased provisions across sessions (GLM: 0.117 ± 0.051, z = 2.305, *p* = 0.021; see Fig. 1).

On an individual level, six birds landed more often in the test compared to both control conditions (0 AGP, 1 BHM, 3 EC, 2 GA; see Table 3 and Table S4). Additionally, four birds landed less often in the empty control (2 AGP, 0 BHM, 0 EC, 2 GA) compared to the landings in the test condition. And five additional birds landed less often in the blocked control compared to the test condition (0 AGP, 2 BHM, 1 EC, 2 GA).

### Effects of species’ socio-ecology

**Fig. 2:**
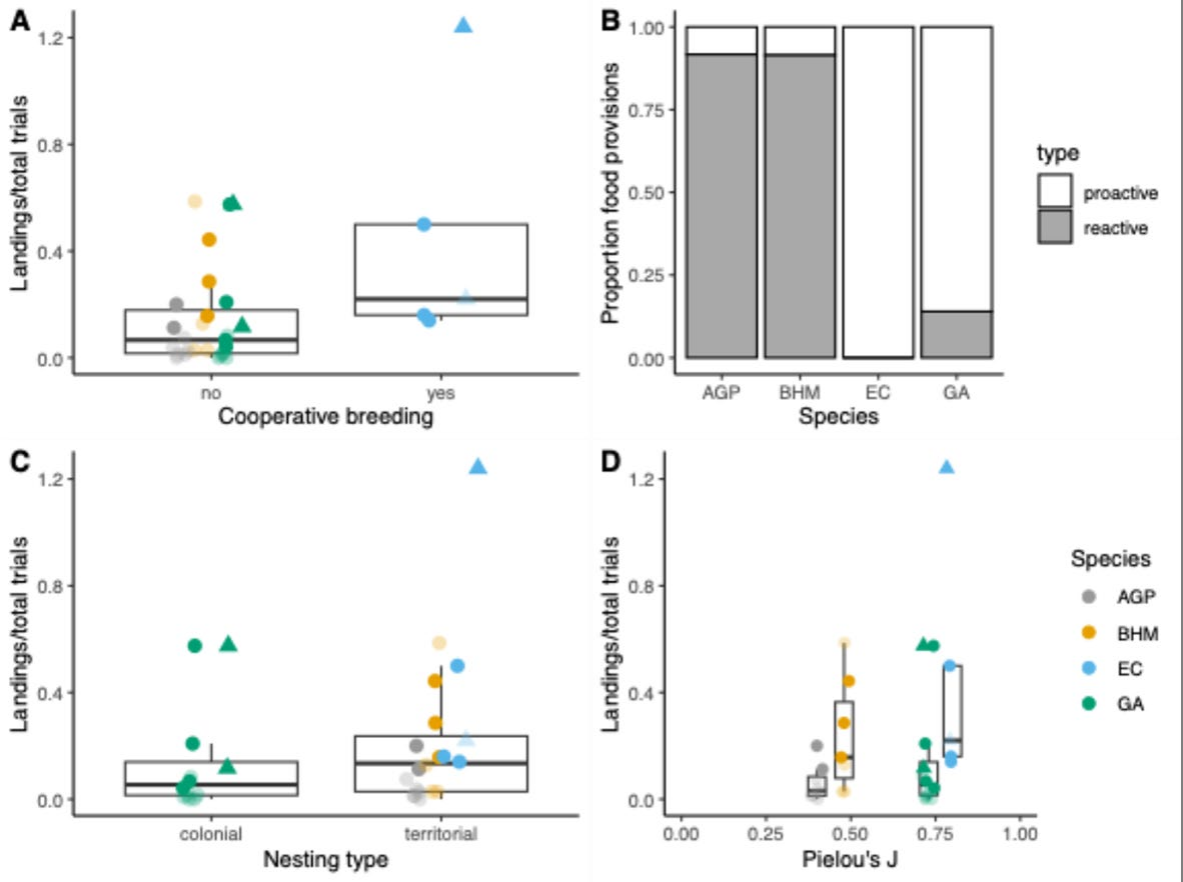
Proportion of landings on provider perch (position 0) in relation to the total trial number per species in the last two test sessions of the group service phase (IV) according to (**A**) Cooperative breeding, (**B**) Proportion of proactive and reactive food provisions per species. (**C**) Nesting type, and (**D**) Social tolerance measured by Pielou’s J. Birds that landed more often in the test compared to either control condition are highlighted in solid points (*N* = 15 birds), birds that did not pass the criterion (*N* = 17) are shown with transparent points and cheaters are indicated with triangles. *AGP* African grey parrots, *BHM* blue-headed macaws, *EC* Eclectus, *GA* Galahs.

Cooperatively breeding species (i.e. EC) landed more often compared to non-cooperatively breeding species (GLM: 4.649 ± 2.754, z = 1.688, *p* = 0.091; see Fig. 2A & Table S5). This fact was mainly driven by the cooperatively breeding males (0.870 ± 0.523; excl. cheater: 0.500) that landed six times as often compared to the non-cooperatively breeding males (0.149 ± 0.188; excl. cheaters: 0.109 ± 0.145). The landing pattern of the females did not differ between cooperatively breeding (0.173 ± 0.042; excl. cheater: 0.150 ± 0.014) and non-cooperative species (0.137 ± 0.193). The pattern does not change when looking only at the individuals that passed the criterion (cooperative: males: 0.870 ± 0.523, females: 0.150 ± 0.014; non-cooperative: males: 0.255 ± 0.219, females: 0.251 ± 0.185, excl. cheaters: 0.137 ± 0.193). The cooperatively breeding EC exhibited exclusively proactive food provisions (100% of all provisions) compared to the three other non-cooperatively breeding species that differed in the proportion of proactive provisions (AGP: 10.0% [birds passing criterion: 8.3%], BHM: 9.1% [birds passing criterion: 8.6%], GA: 86.2% [birds passing criterion: 86.0%]; see Fig. 2B).

Also nesting ecology could serve as an explanation for the observed differences in landing patterns, territorial nesting species landed more often compared to colonially nesting species (i.e. GA; GLM: 4.719 ± 2.251, z = 2.096, p = 0.036; see Fig. 2C). In particular, the territorially nesting males landed three times as often (0.375 ± 0.428; excl. cheaters: 0.230 ± 0.213) than the colonial males (0.129 ± 0.201; excl. cheaters: 0.042 ± 0.034). The females did not differ between the two different nesting styles (colonial: 0.162 ± 0.247; territorial: 0.136 ± 0.153; excl. cheaters: 0.129 ± 0.158). The same pattern became stronger when looking only at the birds that passed the criterion (colonial males: 0.200 ± 0.252; females: 0.392 ± 0.259; territorial males: 0.617 ± 0.425, territorial females: 0.154 ± 0.032). The only colonially nesting species in our study, however, exhibited a high rate of proactive food provisions (see Fig. 2B). Social tolerance could additionally explain species differences in landing patterns (see Fig. 2D). The higher the Pielou’s J’, the more often the birds landed on the provider perch (GLM: 2.736 ± 1.228, z = 0.330, *p* = 0.0258). Overall, it needs to be noted that the effects of cooperative breeding, territorial nesting, and social tolerance could not be tested independently of one another due to the non-balanced species composition in our data set.

### Dyadic provisions

Dyadic characteristics (i.e. affiliation, relatedness and sex) had an effect on the number of provisioning between individuals (full-null comparison: LRT: Chisq = 42.802, df = 20, *p* = 0.002; see Table 3). These characteristics were, however, not species-specific since all interaction terms were non-significant (LRT: species x affiliation: Chisq = 1.573, df = 3, *p* = 0.666, species x sex: Chisq = 10.282, df = 9, *p* = 0.328, species x kinship: Chisq = 4.373, df = 3, *p* = 0.224; see Table S6). Consequently, we fitted a reduced model lacking the interaction terms but retaining species as a control variable (see Table S7). The number of dyadic provisions differed between species (LRT: Chisq = 34.164, df = 3, *p* < 0.001). Furthermore, dyads with a higher affiliation score provided more food to one another (GLMM: 0.640 ± 0.169, z = 3.782, *p* < 0.001). Non-related individuals provided more food to one another than related individuals (GLMM: 1.449 ± 0.527, z = 2.750, *p* = 0.006). We did not observe an effect of sex in this model (LRT: Chisq = 3.849, df = 3, *p* = 0.278).

**Fig. 3:**
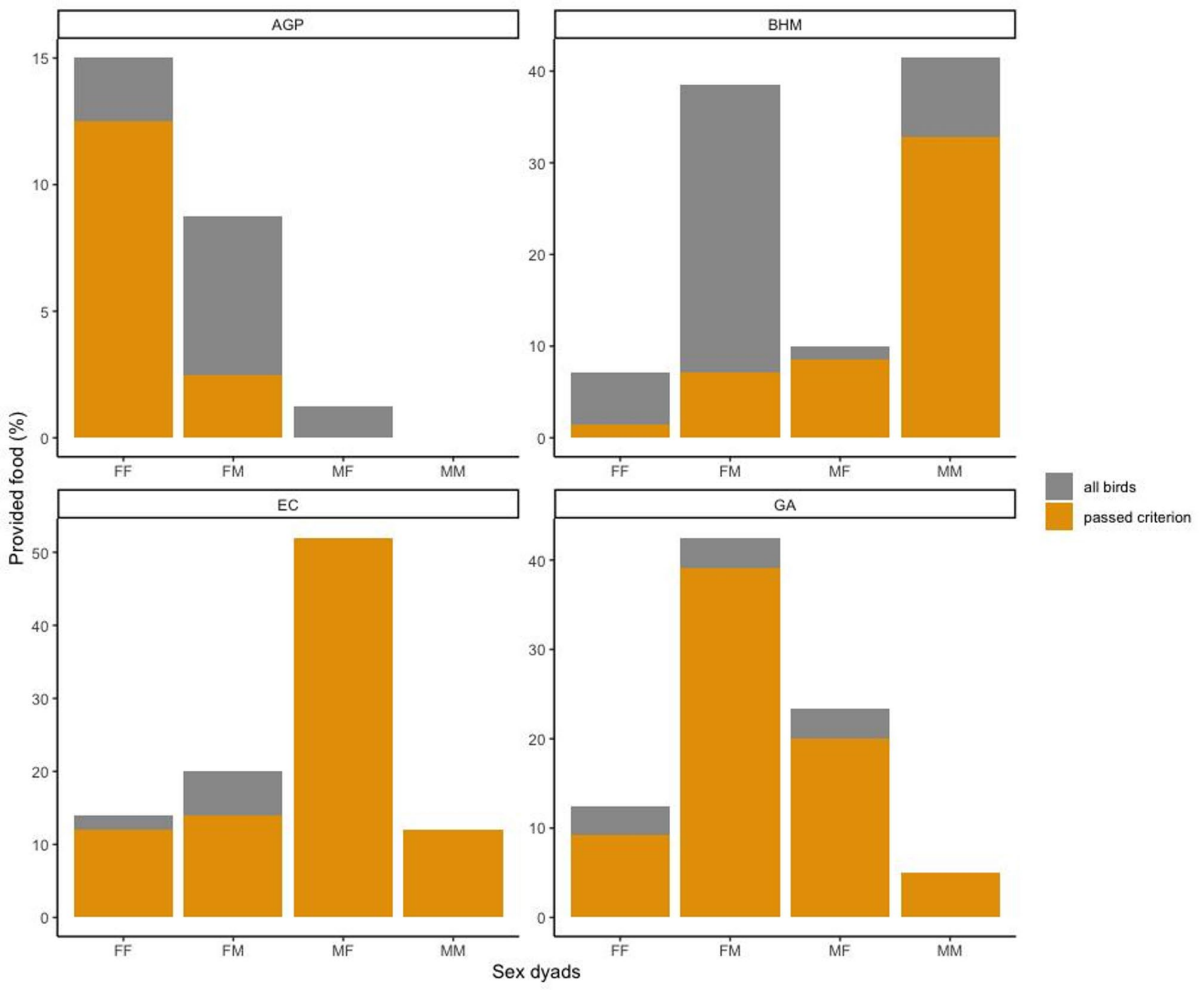
Percentage of successful food provisions in the group service phase (in last two test sessions) plotted per dyadic sex composition for each species. Dyad types: FF = female donor–female receiver, FM = female donor–male receiver, MF = male donor–female receiver, MM = male donor–male receiver. Grey and yellow colours correspond to whether all individuals (*N* = 32) were used in calculating the percentage of food pieces provided or only the individuals that passed the control criterion (*N* = 15), respectively. *AGP* African grey parrots, *BHM* blue-headed macaws, *EC* Eclectus, *GA* Galahs.

The kinship effect on dyadic provisions got weaker, when re-running the analyses including only birds that passed the test criterion; all other effects remain unchanged (see Table S9). And a sex effect emerged, when excluding the four cheaters from the analyses, while the rest of the results remain unchanged (see Table S8). In particular, provisions between males (0.011 ± 0.033 provisions/trials) and from females and males (0.010 ± 0.027) occurred more often compared to provisions between females (0.003 ± 0.011) or from males to females (0.004 ± 0.025; see Fig. 3).

**Table 3:** Overview of results per species.

	Species					
	AGP	BHM	EC		GA	
**Group-level**						
Motivation	→	→	→		→	
Successful provisions	60.7% of all trials 25.0% in S4/5 ↓ across sessions	94.9% of all trials 94.3% in S4/5 → across sessions	99.4% of all trials 98.0% in S4/5 → across sessions		60.5% of all trials 78.3% in S4/5 ↑ across sessions	
Percentage proactive	10.0%	9.1%	100.0%		86.2%	
** Individual-level**						
Control criterion	2/8	3/7	4/5		6/12	
**Dyadic-level**						
Sex	FF > others	MM > others	MF > others		FM > others	
Affiliation	↑	↑		↑		↑
Kinship	Non-related > kin					

↑ = increase/positive effect, → = constant/no effect, ↓ = decrease/negative effect.

## Discussion

As a first assessment of socio-ecological factors that may select for prosociality in parrots, we tested four distantly related parrot species differing in their parental care and nesting ecology in the group service paradigm. We found that all four parrot species provided food to group members, however, the number of such prosocial acts differed between species. Some individuals of each species appeared aware of when food could actually be delivered to others, as apparent from the higher number of landings in the test compared to the two control conditions. Socio-ecological factors could serve as explanations for the observed species differences: the cooperatively breeding eclectus parrots acted more prosocial compared to the non-cooperatively breeding species, territorial nesting species (i.e. BHM, AGP, EC) landed more often compared to colonially nesting species, and social tolerance was associated with higher landing rates. Furthermore, on a dyadic level, affiliation was positively related to prosocial acts, while kinship negatively influenced prosociality.

Compared to the other species tested with the group service paradigm (e.g. rooks: 2%, common ravens: 21%, azure-winged magpie: 98%^20^; bonobos: 2.0%^16^; capuchin monkeys: 3.1%, macaques: 7.7%, marmosets: 60.9% across all sessions^23^) the parrots in our study provided food to others at low (AGP: 25%) to high levels (GA: 78.3%, BHM: 94.3%, EC: 98.0%); thus, suggesting that most parrots species are genuinely prosocial. Amongst the eclectus parrots, almost all birds landed more often in the test compared to the control conditions and exhibited sustained provisioning rates across sessions. These results suggest that eclectus exhibited a genuine prosocial intent^23^. The galahs exhibited increased provisioning rates across sessions and also the blue-headed macaws sustained high provisioning rates throughout sessions. On the contrary, the African grey parrots decreased their provisioning rate across sessions despite landing in almost every trial. Given the low social tolerance observed in the African greys, this result may indicate that individual birds monopolised the apparatus, preventing other birds from landing on the receiver perch. Interestingly, this result is in stark contrast to previous studies that found high levels of prosociality in African grey parrots in a conspecific^32^ and heterospecific^35^ dyadic setting but not in blue-headed macaws^32^. However, it also indicates that prosociality could be task-specific (e.g^34^. see also^20,25^) and might greatly depend on social dynamics (i.e. dominance^56^) that are artificially controlled in dyadic settings. But also individual differences in task understanding or prosociality might play a role as only half of the birds or less (i.e. ¼ of the African greys) clearly discriminated between test and control conditions on an individual level. Also, only a small number of birds (see supplemental materials) attempted, but never succeeded, to gain access to the food reward after landing on the provider perch, suggesting that the remainder birds associated the consequences of landing on the provider perch with the impossibility of gaining food for themselves.

The interspecific differences in prosociality can be explained by socio-ecological factors. The cooperative breeding eclectus landed more often on the perch and thus acted more prosocial than the non-cooperative breeding species in this study. This effect was mostly driven by the males that landed more often than the males of non-cooperatively breeding species. In the wild, male eclectus are helpers during breeding and groups of males provide food for multiple breeding females^43^. Therefore, it was expected that male eclectus acted more prosocial than both female eclectus and the individuals of the other non-cooperative breeding species. Furthermore, all of these provisions were proactive while the number of proactive provisions was lower in the other species. Given that the only cooperatively breeding species in our study exhibited clearly higher prosocial concern than the other species, our data support the cooperative breeding hypothesis^18^. Interestingly, species-specific sex-roles of helpers seem to be the determining factor for prosociality, since in cooperatively-breeding corvids, for example, the majority of prosocial acts were driven by landings of the female helpers instead of males^20^.

We also found that nesting ecology may explain prosocial tendencies between species. Males of territorial nesting species acted more prosocial than males of colonially nesting species while no difference could be found with regard to females. In African grey parrots and eclectus, the females usually defend their nest while the males search for food in social foraging flocks^43,44^ and therefore, male parrots should be more tolerant of other individuals. Interestingly, an opposite effect of nesting ecology was observed in corvids, in which males of colonial nesting species acted more prosocial than males of territorial species^20^. One might hypothesise that this difference between parrots and corvids may be explained by differences in the breeding and feeding biology of the two taxa. In contrast to the eclectus parrots, in which the helpers are usually males^42^in corvids, female helpers are more common. This highlights that differences in breeding biology (i.e. sex-specific roles of defending nest and providing food) need to be considered carefully when deriving predictions across taxa. However, it needs to be noted that, a final conclusion is difficult with the current data on just four species and considering that data on parrot species in the field are very limited.

Overall, it seems that social tolerance best explains the species differences in prosociality. Generally, the species with higher social tolerance also acted more prosocial with high levels of proactive provisions (i.e. eclectus and galah), whereas the African grey parrots exhibited the lowest provisioning rate (i.e. almost no proactive provisions) and exhibited the lowest social tolerance. While these results support the hypothesis that social tolerance is an underlying mechanism facilitating prosociality (self-domestication hypothesis^13^), the blue-headed macaws, do not fit into the prediction. This species showed only moderate social tolerance but high prosociality in the group service phase. Consequently, in addition to general social tolerance that certainly is required as a basis for having others close to a food source, interdependence might be equally important. The interdependency hypothesis states that cooperative interactions are more likely to occur if individuals or groups are relying on one another for support^57^. Accordingly, the prosocial behaviour of the blue-headed macaws, in spite of a low group-level tolerance, might be explained by their dependency on their partner or group during breeding that elevates the need to show a prosocial concern for others. Blue-headed macaws have been observed in small groups^47^ that potentially are more dependent on one another for survival compared to African grey parrots, which are living in bigger groups with fission-fusion dynamics^58^ and, thus, constantly changing group composition.

Dyadic-level variation in prosocial acts were best explained by non-relatedness, affiliation and sex. Successful food provisions were most likely to occur between dyads with higher affiliation scores. This is in line with other studies that found that prosocial acts are often directed towards affiliated partners (see^4^ for a review). In addition, kinship had a strong negative effect on provisions in all species. Provisions were more likely to occur between non-related individuals. This result is surprising given that it contradicts kin selection theory^59^ and is contrary to other studies (e.g^21^) , in which high provisioning rates were observed amongst related individuals. Either other relationship characteristics are more important than kinship bonds in parrots or this effect was goverened by the limited group size in our zoo-housed parrots (i.e., fewer mates available). Also, the sex of the receiver played an important role for prosociality and this effect was markedly different between species. Female to female provisions were most prevalent in the African grey parrots, while the blue-headed macaws provided food mostly from males to males, the eclectus parrots predominantly from males to females, and the female galahs provided most food to males of the group. Differences in breeding biology (i.e. cooperative breeding in eclectus^43^ with males providing for females) but certainly also effects of the limited partner choice, biased sex and kinship compositions of the captive groups might have caused these effects.

There are several limitations to our study that need to be carefully considered. We tested only four species with one group per species, which clearly limits the generalisation power. Furthermore, we tested captive populations, which might result in different underlying motivation to participate in cognitive testing compared to wild populations. Due to a lack of hunger and other stimulation, parrots might be particularly prone to engage in tasks involving food rewards, (i.e. contrafreeloading; e.g^60^). which might explain the high landings rates and participation in the motivational trials compared to other species. Given the artificial group composition in terms of size, sex ratio, age and kinship, effects of dyadic preferences might be biased as the choice of partners was limited. Indeed, previous studies have shown that prosociality is affected by group-specific dynamics that affect social tolerance^16^. In addition, some parrots (2 galahs and 2 eclectus) managed to outsmart the group service paradigm. Our test and control sessions always started with a motivation trial and every sixth trial thereafter also consisted of motivation trials. The four individuals used an alternative strategy and consistently stepped on the provider perch. This resulted in a faster food acquisition during the motivation trials of the control conditions since stepping on the perch would end the regular trial during these sessions. The occurrence of this behaviour might have been facilitated by the presence of a metal bar underneath the seesaw in the enclosure of the two species (see Video 1). Birds could stay in close proximity to the providing perch by sitting next to it and could easily step on and off the perch. Note that it were also some of these birds (see supplemental materials) that tried to cheat most by trying to get the reward themselves after having stepped on the perch in regular trials. Future studies need to ensure that the provider perch is not effortlessly accessible and could additionally reduce the risk of subjects outsmarting the paradigm by interspersing motivation trials less frequently, randomly and unpredictable. Note, however, that if we exclude these cheaters, the results remain similar.

To draw more robust conclusions about the evolutionary drivers of prosociality in parrots, additional groups of each species would ideally have to be tested to reduce the influence of group dynamics (i.e. sex-ratio, kinship, age structure) on prosociality. Future studies need to test many more closely and distantly related parrot species with varying socio-ecological backgrounds (i.e. other cooperative breeding parrots like vasa-parrots^61^ or New Caledonian Parakeets^62^; or colonial nesting species, like Burrowing Parrots^63^ or Monk Parakeets^64^). Large-scale collaborative approaches^65,66^, are a promising way to generate more robust comparative data that will ultimately allow conclusions to be drawn about the evolutionary drivers of cognition.

## Conclusion

This is the first study that tested parrots’ prosocial tendencies using a standardised comparable procedure. The results of this study suggest that cooperative breeding, social tolerance, and a reliance on other group members (i.e. based on affiliation) may be driving factors in the evolution of prosocial behaviour in parrots; thus, providing cautionary support for the cooperative breeding and self-domestication hypothesis but also highlighting the importance of dyadic dependencies based on affiliation as suggested by the interdependence hypothesis.

## Electronic supplementary material

Below is the link to the electronic supplementary material.

Supplementary Material 1

Supplementary Material 2

Supplementary Material 3

Supplementary Material 4

## Data Availability

Data is provided within the supplementary information files.
