# Supplementary material for: Exploring the drivers of inter- and intraspecific differences in prosociality in four parrot species
Source: Sci Rep. 2025 Jul 2;15:23558. doi: 10.1038/s41598-025-04115-z (PMC12222856; doi:10.1038/s41598-025-04115-z)
Supplement: Supplementary file 3 — Supplementary Material 3 [file 41598_2025_4115_MOESM3_ESM.docx]

**Video 1.** Procedure of group service paradigm per species and condition, including behaviour of cheating individuals.
