# Supplementary material for: Exploring the drivers of inter- and intraspecific differences in prosociality in four parrot species
Source: Sci Rep. 2025 Jul 2;15:23558. doi: 10.1038/s41598-025-04115-z (PMC12222856; doi:10.1038/s41598-025-04115-z)
Supplement: Supplementary file 4 — Supplementary Material 4 [file 41598_2025_4115_MOESM4_ESM.pdf]

Supplementary Material

Exploring the drivers of inter- and intraspecific differences in prosociality in four parrot species

Désirée Brucks[a,b,c]*, Nick C.P. Dam[d*], Anastasia Krasheninnikova[a,b], Bethan McGregor[b], Hari Tsivlin[b], Auguste M.P. von Bayern[a,b#] & Jorg J.M. Massen[e,f#]

[a] Max Planck Institute for Biological Intelligence, Seewiesen, Germany
[b] Max-Planck Comparative Cognition Research Station, Loro Parque Fundación, Puerto de la Cruz, Tenerife, Spain
[c] Animal Husbandry and Ethology, Albrecht-Thaer Institute of Agricultural and Horticultural Sciences, Humboldt Universität zu Berlin, Berlin, Germany
[d] Institute of Biology, Leiden University, Leiden, The Netherlands
[e] Animal Behavior & Cognition, Department of Biology, Utrecht University, Utrecht, The Netherlands
[f] Avifauna Bird Park, Alphen aan den Rijn, The Netherlands

[*] contributed equally
[#] shared last authors

Supplementary Procedures

1. Animals and Housing

1.1 African grey parrots and blue-headed macaws

Both parrot species were housed in different aviaries at the Max-Planck Comparative Cognition Research Station in the Loro Parque in Puerto de la Cruz, Tenerife, Spain. Blue-headed macaws (BHM) were housed in an indoor aviary (28.61 m^2) connected to a smaller outdoor area. The aviary of the African grey parrots (AGP) consisted of an indoor part (2 x 7 x 3.6 m) and an outdoor aviary (1.5 x 7 x 3.6 m). Social groups of AGP and BHM consisted of eight and seven individuals, respectively. Both aviaries always had access to an outside area ensuring a natural day-night schedule. Additionally, Arcadia Zoo Bars (Arcadia 54 W Freshwater Pro and Arcadia 54 W D3 Reptile lamp) were used so that the parrots were exposed to sufficient UV light. Both species had access to water *ad libitum* and were daily fed, twice a day, with a mixture of vegetables and fruits. Furthermore, parrots were additionally fed with seeds in the evening.

The AGP were all 7 years old, none of the parrots were mated pairs during the study. The group consisted of three sibling pairs (A: 3 birds, B: 2 birds, C: 3 birds), thus, 25% of birds were related of all possible dyads. The BHM were all 4 years old and likewise did not form mated pairs yet. Two sibling pairs were in the group, resulting in 9.5% related dyads in the group.

1.2 Galahs and eclectus

The eclectus parrots (EC) and galahs (GA) were housed at Avifauna bird park in Alphen aan den Rijn, the Netherlands. Both parrot species were housed in the same enclosure together with three cockatiels (*Nymphicus hollandicus*). The enclosure consisted of eight indoor compartments (1.3 x 2.25 x 3 m) connected to an outdoor aviary (11 x 7 x 4 m). Social groups of EC and GA consisted of five and 19 individuals, respectively. Enrichment was provided within the enclosure, such as ropes, perches,

branches, nest boxes, plants and trees. Parrots had access to water *ad libitum* and were daily fed with parrot pellets, mashed vegetables, nuts, fruit, and seeds. The EC were more dominant than the GA. Therefore, we trained and tested the GA while the EC were not present in the aviary. Both species participated in a free flight bird demonstration. One of the GA (Pecunia) collected money from visitors after the bird demonstration and was therefore not always present in the aviary during our experiment.

The GA were 6.11 ± 3.36 years old (range: 3-13 yrs.) with one mated pair (Zilver man & Zilver pop; see Table S4) with three birds as their offspring and two siblings from another clutch. In total, the group consisted of 23 sibling pairs (15.4 % of all dyads) and 6 parent-offspring dyads (9.2 % of all dyads). The EC were 5.00 ± 1.00 years (range: 4-6 yrs.) with no mated pairs but four siblings as well as one unrelated bird (60% of all dyads related).

2. Apparatus

We used a wooden apparatus that was attached to the outside of the aviary, similar to Horn et al. (2016). The apparatus consisted of a wooden board that was attached to the outside of the aviary. The board could be moved up and down using a seesaw mechanism. Subjects could operate the seesaw by stepping or landing on a provisioning perch inside the aviary, which was attached to one side of the wooden board by two sticks that extended through the wire mesh of the aviary (see Fig. S1A). In the starting position, the perch pointed upwards and therefore the wooden board was pointing downwards, by balancing the seesaw mechanism (see Fig. S1B). Therefore, when a parrot landed on the provisioning perch, the seesaw moved downwards (see Fig. S1B). Consequently, the apparatus automatically returned to the starting position after the parrot left the perch.

Food (AGP/BHM: sunflower seed, EC/GA: blueberry) was either placed in position 0 or position 1 on the wooden board. Position 0 was located in front of the provisioning perch while position 1 was located on the other side of the board and was unreachable from the perch. Food could slide down from either position towards the mesh through two U-shaped metal channels with barriers at either end after an individual had landed on the perch. If a subject landed on the perch, it could either deliver food to itself (if food was positioned in position 0) or to a group member, depending on the position in which the food was placed. If the food was placed in position 1, the food was out of reach of the perch and only another bird could collect the food. Group members could collect the food either by landing on a second perch that was attached to the wire mesh (in case for the AGP and BHM) at position 1 or by landing on a metal bar in front of the wire mesh (in the case of the GA and EC).

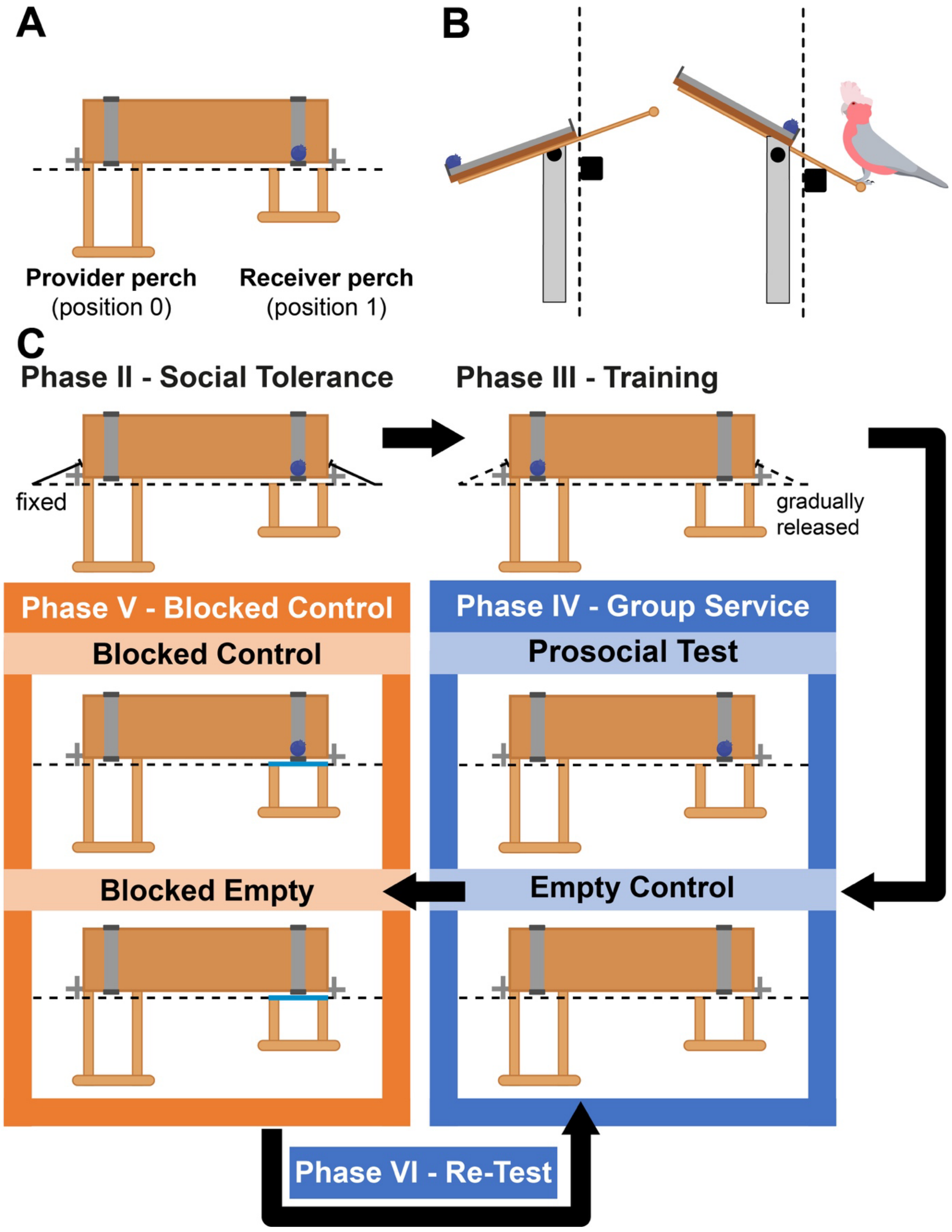

Fig. S1 A) Schematic representation of the apparatus with landing positions, B) seesaw mechanism operating apparatus when bird lands on provider perch, C) overview and timeline of different phases in the group service paradigm .

3. Group Service Paradigm Procedure

We habituated, trained and tested all species in a fixed sequence over six consecutive phases, similar to Horn et al. (2016; see Table S1). However, we did not include the first habituation phase (phase 0, see below) used in Horn et al. (2016) for the GA and EC since these parrots exhibit less object-related

neophobia than corvids (personal observations). The EC were already trained and tested until phase IV (see below) but could not complete the experiment due to Covid-19 restrictions. Therefore, we repeated the experiment for this species. We trained and tested as many individuals per species as possible. The number of subjects tested depended on the number of parrots that passed the training and habituation criteria (see below). Consequently, the number of trials during the test and control sessions per species was determined by the number of birds that passed these criteria and was calculated as five times the number of subjects that passed the criteria. Birds were tested during the same daily time for all conditions for each species, so that motivation levels were comparable between conditions. All behaviours during sessions were noted by a researcher and all test and control sessions (phase IV, V and VI) were recorded on camera (Canon Legria HF R606 camcorder) for confirmation in later analysis.

Table S1. Overview of training and test procedure

**Phase**		**Mechanism**	**Food**	**Sessions**
0	Habituation to apparatus	Fixed	Bowl at position 0	Until all birds have fed from bowl 5x
I	Habituation to procedure	Fixed	Alternating between sessions	Until all birds have taken 10 food items across 5 sessions
II	Social tolerance	Fixed	Position 1	2 sessions
III	Training	Gradually released	Position 0	Until all birds have taken 10 seeds across 5 sessions
IV	***Group Service***			
	1) Prosocial Test	Released	Position 1	5 sessions
	2) Empty Control	Released	No food, only movement	5 sessions
V	***Blocked Control***			
	1) Blocked Control	Released	Position 1, blocked by mesh	5 sessions
	2) Blocked Empty	Released	No food, blocked by mesh	5 sessions
VI	***Re-Test*** (after break)			
	1) Prosocial Test	Released	Position 1	2 sessions
	2) Empty Control	Released	No food, only movement	2 sessions

Phase 0: habituation to the apparatus (only for AGP and BHM)

The seesaw mechanism was fixed in a way that landing on the perch did not cause any movement of the apparatus, to avoid additional neophobic reactions. For the AGP and BHM, a bowl filled with food, was placed at the provider position (pos 0) and it was noted which bird landed and consumed food within 30 min. Once all birds had fed from the bowl at least five times, the next step was initiated.

Phase I: habituation to the procedure

We trained the parrots to understand that food could be obtained from the apparatus once the experimenter placed a piece of food on the wooden board. The apparatus was fixed so that the perch was pointed downwards by holding the board with one hand. This allowed the food to automatically slide down towards the wire mesh after being placed on the board. Subjects were trained to receive food from both positions (provider and receiver position). Therefore, food was placed in either the provider or receiver position on alternate days. A trial ended when a parrot obtained a piece of food or after a maximum of two minutes. To account for the different group sizes, the number of trials was adjusted to the group size (i.e. number of trials = 5 x group size; AFG: 40 trials, BHM: 35 trials, E: 25 trials, G: 75

trials (later adjusted to the number of birds passing the criterion), similar to Horn et al. (2016, 2020)). For the GA, we used a more flexible approach as we did not plan to train all 19 GA, but rather as many individuals as possible. Therefore, sessions ended based on how many individuals were willing to come to the apparatus and the observations and judgement of the experimenter (ND). However, a session ended when no subject stepped on the provisioning perch after three consecutive trials. Sessions were repeated until all birds had taken at least 10 food items in 5 sessions. If a bird monopolised the apparatus, he/she was separated from the group for the next session. All birds except for four GA reached the criterion.

Phase II: social tolerance test
In this phase, we assessed the social tolerance by measuring the evenness of food distribution within each group. All procedures were the same as in phase I and the apparatus was fixed in a downward position. However, we only placed food at the receiver position (pos 1). The number of trials per session was 35, 40, 25 and 75 for the AGP, BHM, EC and GA, respectively. The social tolerance test was conducted twice, on two consecutive days, for each species. We recorded which individual received each piece of food.

Phase III: apparatus training
The parrots were trained to operate the apparatus themselves by stepping on the provider perch (position 0). In case of the AGP and BHM, we gradually released the seesaw mechanism across sessions to habituate the individuals to the movement of the seesaw. In contrast, the seesaw mechanism was immediately fully released for the GA and EC. Food was always provided at the provider position (pos 0) and could be obtained by stepping on the provisioning perch (pos 0). Subsequently, the subject had to step off the perch after obtaining the food reward. Three GA (Miss G, Dali and Lickytung) were afraid of the board's movement. Therefore, we used the same gradual procedure as for the AGP and BHM for these three individuals. All other procedures were the same as in the previous phases. A parrot passed this phase when she/he had obtained more than ten pieces of food in at least five sessions by stepping on and off the provider perch. Dominant individuals monopolising the apparatus were either excluded from the study (i.e. one GA attacked any bird that came close to the apparatus) or selectively separated from the group to allow low-ranking birds to approach the apparatus. Note that dominant birds were only separated temporarily from the group in phase I and phase III so that all subjects had the opportunity to learn about the mechanism of the seesaw. During the remaining test phases all subjects were present. All birds except for three GA reached the criterion of this phase.

Phase IV: group service phase
The group service phase consisted of five test sessions and five empty control sessions, alternating between days. During regular test trials, the experimenter placed food on the receiver perch (pos 1). Therefore, a parrot that stepped on the provider perch (pos 0) could only provide food to one group member. In contrast, during regular control trials, no food was placed at the receiver position (pos 1), but instead we pretended to place a piece of food on the board using the same hand movements as in the test condition. We conducted 40 trials with the AGP, 35 trials with the BHM, 60 trials with the GA, and 25 trials with the EC. Both the test and control sessions started with a motivation trial, in which food was placed at the provider position (pos 0). Furthermore, there was a motivational trial after every fifth trial in both regular and control sessions (see Table S1).

Food was only placed down on the board when a parrot had stepped off the provider perch. A new trial was started as soon as a food reward was successfully provided or when two minutes had passed without a bird landing. All other procedures were the same as in the previous phases. In the case of the GA, untrained individuals (N = 5) were still present in the aviary and therefore able to participate, but we excluded these individuals from the analyses (except for dyadic analyses). For each trial, we recorded

which individual provided the food by stepping on the perch and which individual obtained the food (only in the case of the test condition). We also recorded which individuals stepped on the provider perch and which individuals sat in front of the receiver position (pos 1).

Phase V: blocked phase
This phase consisted of five blocked control and five blocked empty control sessions, alternating between days. In all sessions, we blocked the access to the food at the receiver position (pos 1) with a piece of Plexiglas. This controlled for the possibility that subjects stepped on the provider perch due to the presence of food. As an additional control, we did not place any food at the receiver position (pos 1) during the blocked empty control sessions, similar to the empty control of phase IV. Parrots still had access to the food at the provider position (pos 0) during motivation trials. The procedure and sequence of sessions and trials were the same as in phase IV. The same variables were coded as in phase IV.

Phase VI: repeated phase
We repeated the group service phase ten (AGP), five (GA) and two weeks (EC) after completing the blocked phase V. The BHM were not tested in the re-test phase due to time constraints. Two test and two control sessions were conducted, again one alternate days (as in phase IV). The same variables were coded as in phase IV.

Supplementary Analyses

4.1 Social Tolerance
Social tolerance within each group was scored based on the evenness of the distributed food in phase II. Similar to previous studies (Horn et al., 2020), we calculated an averaged Pielou's J score for both sessions in phase II. This score ranges from 0 (complete inequality) to 1 (complete equal distribution).

4.2 General model diagnostics
We used the package *lme4* (Bates et al., 2014) for running the GLMMs and GLMs and reverted to using *pscl* (Zeileis et al., 2008) and *glmmTMB* (Brooks et al., 2017) for zero-inflated models. Model assumptions (i.e. collinearity, distribution of random effects, overdispersion, zero-inflation) were checked for each model separately. Collinearity was assessed using Variance Inflation Factors (VIF; *vif*-function in 'car' package (Fox & Weisberg, 2019)) based on reduced models lacking any interaction terms. To test for the effects of the predictors, we always compared the full model to a null model (intercept only, but with the same random effects structure) using Likelihood ratio tests (LRT) as a means to avoid cryptic multiple testing (Schielzeth & Forstmeier, 2009). Model stability was assessed by dropping the levels of random effects one at a time and comparing the estimates to the models based on the full data. Confidence intervals were obtained using the function *bootMer* (N = 1000 bootstraps).

Models:
1) To determine whether the birds showed sustained interest in the task, we analysed each species' landing frequency in the motivational trials across all sessions separately. We fitted a generalised linear mixed model (GLMM) with a binomial error distribution and a logit-link function. Number of landings in the motivational trials was set as response variable. To account for a fixed upper limit of motivational trials per session, we used the *cbind*-function and included the number of landings and the total number of motivational trials as a response matrix. Sex (factor: female, male), and an interaction term between condition (factor: test, blocked, empty) and session (numeric: 1-5) were included as predictors. Bird ID was used as random effect and condition, session, and an interaction between condition and session were included as random slopes.

2) To assess whether landing patterns differed between test and control conditions at the individual level, we ran separate Chi-square tests.
3) The development of prosocial acts across the test sessions was analysed using GLMs with a poisson error distribution for each species separately. We set the number of successful food provisions as response variable and session number (numeric: 1-5) as predictor.
4) For assessing factors that might explain species differences in prosociality, we ran a generalised linear model (GLM) accounting for zero-inflation with a negative binomial error distribution. The model was once run including all birds that passed the training criterion in phase III (N = 32 birds) and once only including birds that landed significantly more often in the group service test compared to either of the control conditions (based on the individual analysis in step 2; N = 15 birds). To rule out that cheaters were driving the results, we additionally ran a model excluding the four cheating birds (see below for definition of cheating behaviour). The number of landings on the provider perch was set as response variable and cooperative breeding (factor: yes, no), nesting type (factor: colonial, territorial), and social tolerance (z-transformed) were set as fixed factors. To control for the different group sizes of the species and the resulting differences in trial numbers per session (e.g. EC 25 tests trials/sessions vs. GA 60 test trials/session), we included an offset term of trial number (log-transformed).
5) To assess whether dyadic characteristics (i.e. affiliation, relatedness and sex) explain differences in dyadic provisioning we ran a GLMM with a negative binomial distribution to account for zero-inflation. Number of provisions per dyad was used as response variable and sex, kinship (factor: yes, no) and affiliation score (z-transformed) in interaction with species were set as predictors. Trial number (log-transformed) was included as offset-term. We included provider nested within dyad as random effect and an additional random effect with provider and affiliation as random slope.

Additional Results

5.1 Phase II – Social tolerance

The cooperatively breeding species showed a higher social tolerance in Phase II (Pielou's J' = 0.796) compared to the non-cooperatively breeding species (mean Pielou's J' ± SD = 0.568 ± 0.153). Furthermore, the colonial nesting species exhibited a higher social tolerance (Pielou's J' = 0.731) than territorial nesting species (mean Pielou's J' ± SD = 0.526 ± 0.164).

5.2 Phase IV – Prosocial behaviour

Landings in motivational trials

Overdispersion was not present in any model (all dispersion parameters < 1.16). All random effects showed a symmetrical distribution. All VIFs were smaller than 1.31, accordingly, collinearity was not a problem. The models were generally of acceptable stability, only the model for the AG showed an instability for the sex-effect (see Table S3).

Participation in the motivational trials was consistently high for the majority of species with the exception of the AG that participated less in the motivational trials in the empty control but equally often in the other test conditions (see Table S2).

Table S2. Percentage of motivational trials with landings across the last two sessions per species.

Species	Trials	Test	Empty	Blocked	Re-Test	Re-Control
AGP	18	94%	67%	100%	94%	100%
BHM	16	100%	100%	100%	NA	NA
EC	12	100%	100%	100%	92%	100%
GA[a]	26	85%	85%	100%	85%	96%
**Mean ± SE**		94.75±3.54	88.00±7.84	100.00±0.00	90.33±2.73	98.67±1.33

[a] including only birds that passed phase III

We found no observable effects of session or condition on the number of food items obtained in the motivational trials (full-null comparison - LRT: AG: Chisq = 7.678, df = 6, p = 0.263; BHM: Chisq = 4.987, df = 6, p = 0.545; EC: Chisq = 5.181, df = 6, p = 0.521; GA: Chisq = 9.500, df = 6, p = 0.147); accordingly, the number of landings was constant across test sessions for all species (see Table S3). Furthermore, in the GA and EC both sexes obtained equal numbers of food rewards in the motivational trials. Male AG tended to obtain less food, while male BHM tended to obtain more food in the motivational trials compared to the females.

Table S3. Summary of results and measures for model stability (when excluding one level of the random effect at a time and comparing the estimates to the original estimates from the full model) from the full models with number of food items obtained in the motivational trials as response variable per species.

Term	Estimate ± SE	z	Chisq	df	p-value	Min.	Max.
African grey							
Intercept	-3.316 ± 1.476	-2.246			[1]	-3.714	-1.880
Session[2]	-1.030 ± 0.829	-1.243			[1]	-1.302	-0.287
Condition (blocked)	1.199 ± 1.130	1.061			[1]	-0.084	1.490
Condition (empty)	0.858 ± 1.005	0.854			[1]	-1.483	0.959
Sex (male)	-2.367 ± 0.984	-2.404	2.961	1	0.085	-19.313	0.679
Session:Condition(blocked)	0.795 ± 0.779	1.020	1.658	2	0.431	0.175	1.131
Session:Condition(empty)	0.356 ± 0.684	0.520	1.658	2	0.431	-1.404	0.384
Blue-headed macaw							
Intercept	-2.871 ± 0.666	-4.311			[1]	-3.496	-2.211
Session[2]	-0.116 ±0.308	-0.377			[1]	-0.360	0.080
Condition (blocked)	-1.034 ± 0.862	-1.200			[1]	-1.356	-0.381
Condition (empty)	-0.185 ± 0.371	-0.498			[1]	-0.310	-0.066
Sex (male)	1.422 ± 0.880	1.616	3.513	1	0.061	0.717	1.931
Session:Condition(blocked)	0.026 ± 0.578	0.044	0.010	2	0.995	-0.281	0.380
Session:Condition(empty)	0.036 ± 0.362	0.099	0.010	2	0.995	-0.261	0.510
Eclectus							
Intercept	-3.620 ± 1.509	-2.399			[1]	-4.608	-2.417
Session[2]	0.819 ± 0.743	1.102			[1]	0.025	1.050
Condition (blocked)	-1.275 ± 2.148	-0.594			[1]	-3.238	0.230
Condition (empty)	-2.652 ± 3.250	-0.816			[1]	-3.889	0.024
Sex (male)	1.940 ± 1.552	1.250	1.585	1	0.208	0.728	4.443
Session:Condition(blocked)	0.453 ± 1.659	0.273	0.114	2	0.945	-0.922	1.882
Session:Condition(empty)	0.294 ± 2.976	0.099	0.114	2	0.945	-1.491	0.522
Galah							
Intercept	-3.269 ± 0.404	-8.090			[1]	-3.426	-2.917
Session[2]	-0.218 ± 0.273	-0.799			[1]	-0.346	-0.117
Condition (blocked)	-1.464 ± 0.769	-1.903			[1]	-1.880	-1.104
Condition (empty)	-1.100 ± 0.773	-1.424			[1]	-1.540	-0.718
Sex (male)	0.737 ± 0.519	1.421	1.919	1	0.166	0.265	1.177
Session:Condition(blocked)	0.425 ± 0.486	0.876	0.982	2	0.612	0.131	0.758
Session:Condition(empty)	-0.137 ± 0.513	-0.267	0.982	2	0.612	-0.335	0.191

[1] not shown due to limited interpretability

[2] z-transformed to a mean of zero and a standard deviation of one

Landings across conditions

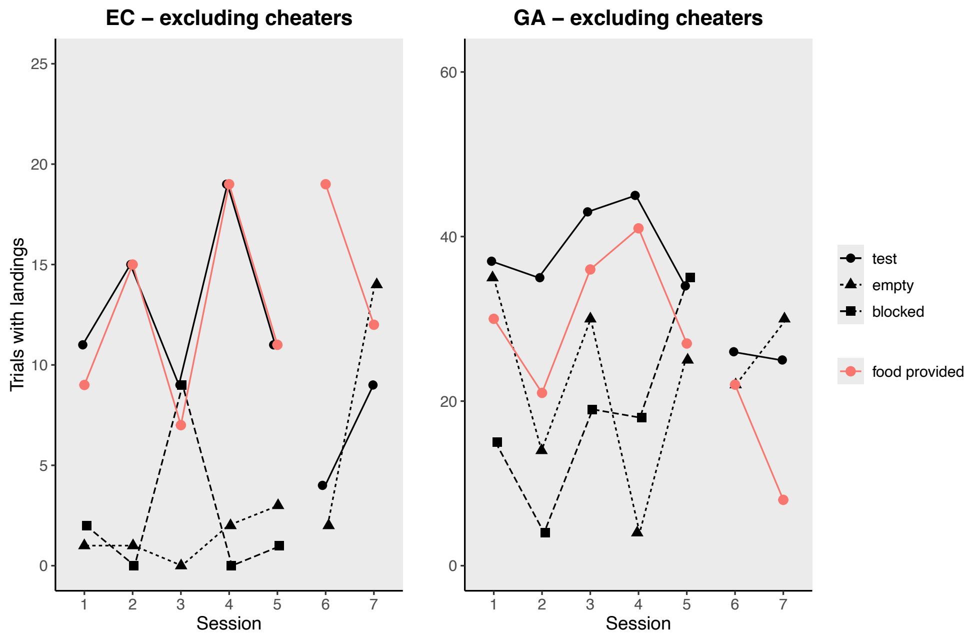

Figure S2. Trials with landings for Eclectus and Galahs when excluding the four cheating birds.

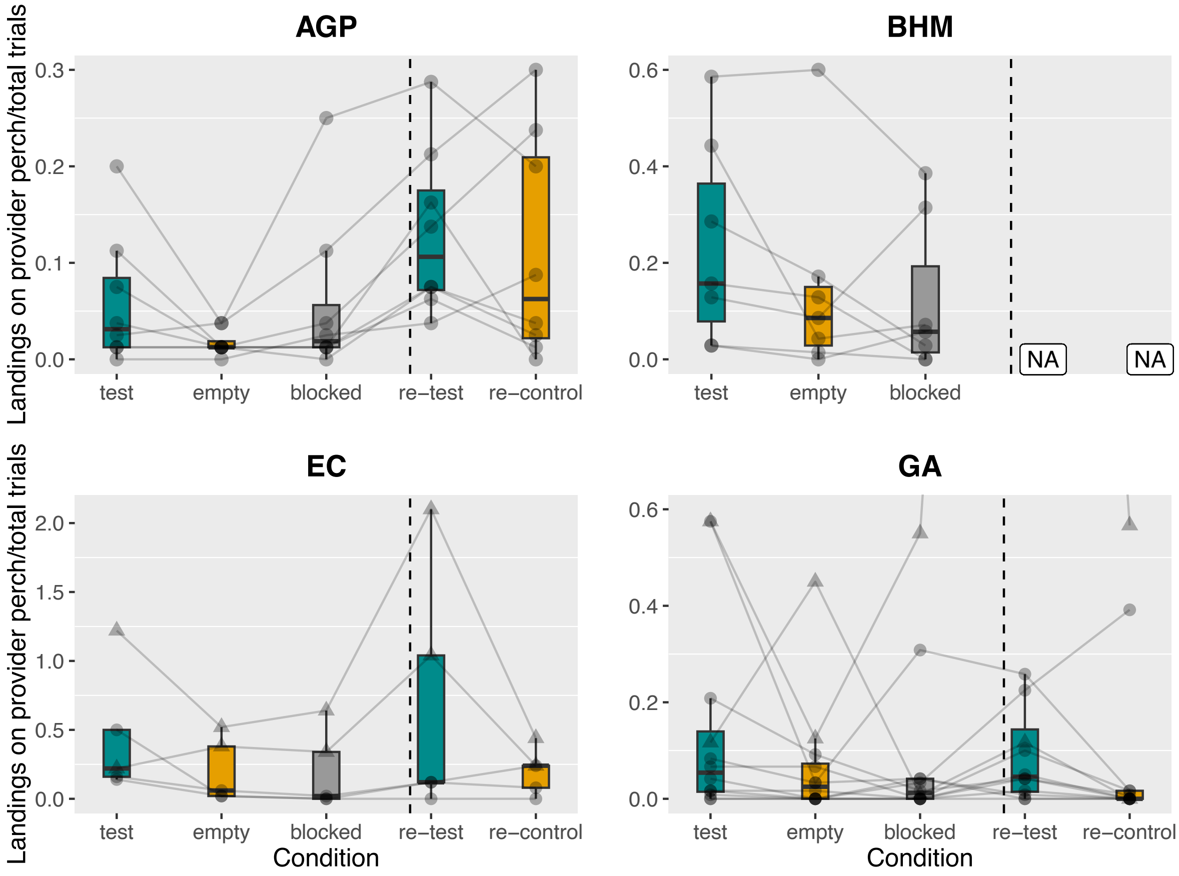

Figure S3. Number of landings on provider perch in the last two sessions in phase IV-VI. Grey points indicate raw data from individual birds; triangles depict cheating individuals (only EC and GA). Points and lines indicate individual landing patterns. Note that for the Galahs, an individual data point is cut off in the re-test condition (3.77).

5.3 Individual level analyses

Table S4. Number of provided and received food in the test phase (last two sessions) and number of landings on the provisioning perch in the test and control phases (last two sessions) per individual.

				GSP test – phase IV		Landings phase IV & V			Landings phase VI	
**Species**	**Name**	**Age[1]**	**Sex**	**provided**	**received**	**test**	**empty**	**blocked**	**test**	**empty**
African grey	Bella[A]	7	F	3	0	6	1	1	6	3
	Jack[B]	7	M	1	7	1	1	1	3	7
	Jelo[B]	7	F	0	0	0	0	2	13	0*
	Kimmi[C]	7	F	2	5	3	1	1	11	19
	**Lizzy**[A]	7	F	7	2	9	1*	3	17	24
	**Nikki**[B]	7	F	5	6	16	3*	9	23	16
	Nina[A]	7	F	2	0	2	3	20	6	2
	Sensei[C]	7	M	0	0	1	1	0	0	0
Blue-headed macaw	**Andromeda**	4	F	4	5	11	9	0*	NA	NA
	Callisto[D]	4	F	1	0	2	0	4	NA	NA
	Lupita[D]	4	F	1	0	2	1	0	NA	NA
	**Mars**[E]	4	M	23	14	31	3*	5*	NA	NA
	**Mercury**[E]	4	M	8	19	20	12	2*	NA	NA
	Neptune	4	F	24	9	41	42	27	NA	NA
	Saturn	4	M	5	19	9	6	22	NA	NA
Eclectus	**Agent Orange**[F,x]	5	M	15	16	62	26*	32*	105	22*
	**Red**[F]	6	M	17	0	25	1*	0*	6	4
	**Blue**[F]	4	F	7	0	7	1*	0*	0	0
	Purple[x]	4	F	4	32	11	19	17	52	12*
	**Rainbow**[F]	6	F	6	1	8	3	1*	6	12
Galah	**Boss**	13	M	4	5	8	8	0*	12	2*
	**Miss G**[G]	4	F	37	19	69	4*	37*	31	2*
	Dali	4	F	2	0	2	2	0	0	0
	Pinball[G]	4	F	1	1	1	0	0	6	0*
	Terrence[H]	7	F	2	4	10	4	5	1	0
	**Angry Bird**[x]	4	M	5	24	14	54	1*	14	0*
	Groot[H]	5	M	1	1	2	0	0	5	0*
	**Lickytung**[I]	3	F	21	13	25	11	2*	5	1
	**Terror Jaap**[I,x]	3	M	21	26	69	15*	66	452	68*
	Zilver pop[H]	11	F	0	0	0	0	0	2	0
	**Zilver man**[H]	11	M	0	0	5	0*	5	0	0
	Lord Burb[H]	5	M	0	0	0	0	4	27	47

[1] age in years
[A- I] indicate kinship
[x] repeated patterns of landings and is thus labelled as a 'cheater'
Birds that landed more often in test compared to either control in phase IV & V are highlighted in bold
* $p < 0.05$ based on Chisq-Tests

5.4. Behaviour following landing on provider perch

As an additional variable to assess the birds' understanding of the group service paradigm, we analysed the birds' behaviour following the landing on the provider perch in the two last test sessions in Phase IV. We coded whether the birds tried to get access to the food reward by reaching over (i.e. covering at least half of the distance) to the receiver perch within three seconds after landing on the provider perch.

In general, it happened rarely that birds tried to get the food reward themselves after having landed on the provider perch; thus, suggesting that the birds understood that they could not obtain the food reward themselves. In particular, the cheating individuals tried to gain access to the food reward (see Table S5), although, also on a low level.

Table S5. Behaviour following landing on provider perch per species.

Species	# of landings on provider perch	# of reaching to receiver side	Proportion of reaching by cheating individuals
AGP	38	1	NA
BHM*	79	10	NA
EC	113	2	1.00
GA	213	33	0.73

*Session 3 was coded instead of session 5 due to a corrupted video file.

5.5. Comparative analyses

The initial model was zero-inflated and overdispersed (dispersion parameter = 22.4), consequently, we fitted a GLM accounting for zero-inflation with a negative binomial error distribution using the function *zeroinfl* from the pscl package. Collinearity was an issue due to the non-balanced species composition within our sample (VIF < 50.7); we kept this limitation in mind when interpreting the results.

Table S6. Summary of results (including all 32 birds) from the full model with number of landings on provider perch as response variable.

Term	Estimate	SE	z-value	Lower CI	Upper CI	p-value
Intercept	-4.1006	1.0225	-4.010	-6.105	-2.097	[1]
Cooperation (yes)	-4.6493	2.7544	-1.688	-10.048	0.749	0.091
Nesting (territorial)	4.7190	2.2511	2.096	0.307	9.131	0.036
z.pielou[2]	2.7363	1.2277	2.229	0.330	5.143	0.026

[1] not shown due to limited interpretability
[2] z-transformed to a mean of zero and a standard deviation of one

5.5.1. Analyses excluding cheaters

Excluding the four cheaters from the comparative model did not change the results (see Table S7).

Table S7. Summary of results (excluding cheaters, N = 28 birds) from the full model with number of landings on provider perch as response variable.

Term	Estimate	SE	z-value	p-value
Intercept	-4.4371	1.0295	-4.310	[1]
Cooperation (yes)	-5.1675	2.7748	-1.862	0.063
Nesting (territorial)	5.0554	2.2454	2.251	0.024
z.pielou[2]	2.7361	1.2217	2.240	0.025

[1] not shown due to limited interpretability
[2] z-transformed to a mean of zero and a standard deviation of one

5.6. Dyadic analyses

The model was zero-inflated when using a poisson error distribution. Therefore, we used a negative binomial error distribution instead of a poisson distribution using the 'glmmTMB' package. Overdispersion was not an issue in the model (dispersion parameter = 0.99). Collinearity was not a problem either (all VIFs < 1.27). The random effect did show a symmetrical distribution. We used a 'bobyqa' optimizer (quadratic approximation) with a maximum of 200,000 iterations to deal with a convergence error in the model.

Table S8. Summary of full model results (including all 424 dyads) from the full model with number of prosocial acts as response variable and dyadic characteristics as predictors.

Term	Estimate	SE	Z	Chisq	df	p
Intercept	-2.389	1.001	-2.387			[1]
Species (GA)	-3.402	1.193	-2.851			
Species (BHM)	-1.631	1.382	-1.181			
Species (AGP)	-1.470	1.241	-1.185			
Kinship (yes)	-3.403	1.660	-2.050			
Sex (FM)	2.598	1.706	1.522			
Sex (MF)	1.521	1.425	1.067			
Sex (MM)	4.653	2.332	1.995			
Affiliation[2]	0.289	0.557	0.519			
speciesGA:kinshipyes	0.866	1.872	0.462			
speciesBHM:kinshipyes	3.746	1.928	1.943			
speciesAGP:kinshipyes	1.230	2.158	0.570	4.373	3	0.224
speciesGA:sexFM	-2.07	1.761	-1.175			
speciesBHM:sexFM	-1.659	1.937	-0.856			
speciesAGP:sexFM	-2.564	1.903	-1.347			
speciesGA:sexMF	-1.028	1.609	-0.639			
speciesBHM:sexMF	-1.359	1.982	-0.686			
speciesAGP:sexMF	-3.349	2.178	-1.538			
speciesGA:sexMM	-4.524	2.460	-1.839			
speciesBHM:sexMM	-2.520	2.620	-0.962			
speciesAGP:sexMM	-31.619	487861.598	0.000	10.282	9	0.328
speciesGA:z.aff[2]	0.599	0.596	1.004			
speciesBHM:z.aff[2]	0.106	0.697	0.152			
speciesAGP:z.aff[2]	0.406	0.824	0.493	1.573	3	0.666

[1] not shown due to limited interpretability
[2] z-transformed to a mean of zero and a standard deviation of one

Table S9. Summary of reduced model results (including all 424 dyads) from the full model with number of prosocial acts as response variable and dyadic characteristics as predictors.

Term	Estimate	SE	Z	Chisq	df	p-value
Intercept	-5.624	0.600	-9.373			[1]
Species (BHM)	1.419	0.825	1.720			
Species (EC)	2.957	0.934	3.167			
Species (GA)	-1.576	0.682	-2.309	34.164	3	<0.001
Kinship (yes)	-1.449	0.527	-2.750	8.420	1	0.004
Sex (FM)	0.581	0.437	1.329			
Sex (MF)	0.234	0.611	0.383			
Sex (MM)	1.037	0.664	1.562	3.849	3	0.278
Affiliation[2]	0.640	0.169	3.782	12.948	1	<0.001

[1] not shown due to limited interpretability
[2] z-transformed to a mean of zero and a standard deviation of one

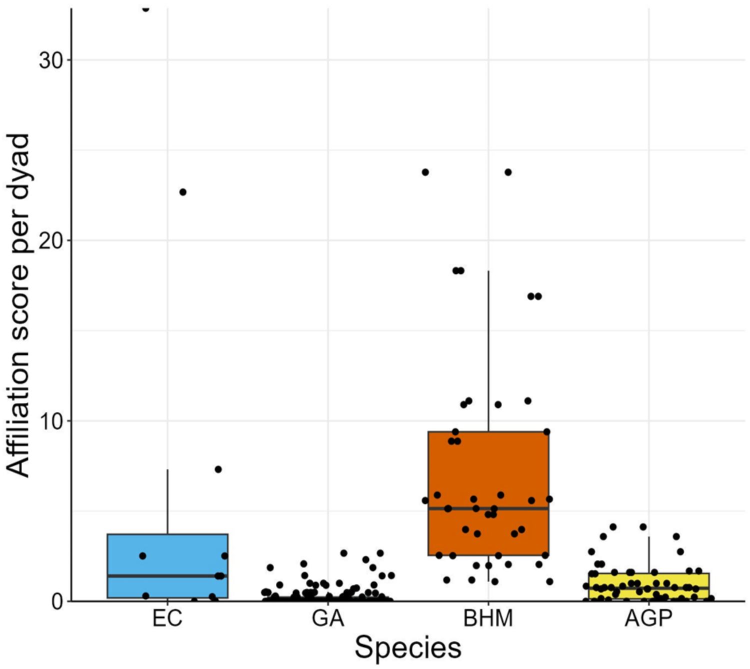

Figure S4. Affiliation score per dyad compared between the different species with boxplots. Medians, interquartile ranges as well as minima and maxima are represented by horizontal lines, boxes and whiskers, respectively. Each dot represents a dyad: Eclectus (EC) N = 12, Galah (GA) N = 170, Blue-headed macaw (BHM) N = 42, African Grey (AGP) N = 56.

5.6.1 Excluding cheaters

The model was found to be zero-inflated, consequently, we used a negative binomial error distribution. The full model had a better fit than their respective null model (LRT: Chisq = 19.877, df = 5, p = 0.001).

Table S10. Summary of model results (excluding cheaters, N = 382 dyads) from the full model with number of prosocial acts as response variable and dyadic characteristics as predictors.

**Term**	**Estimate**	**SE**	**Z**	**Chisq**	**df**	**p**
Intercept	-5.559	0.588	-9.448			[1]
Species (BHM)	1.363	0.801	1.702			
Species (EC)	3.049	1.060	2.876			
Species (GA)	-1.743	0.680	-2.564	29.796	3	<0.001
Kinship (yes)	-1.360	0.528	-2.578	7.340	1	0.007
Sex (FM)	0.635	0.424	1.497			
Sex (MF)	-0.508	0.679	-0.748			
Sex (MM)	0.987	0.704	1.403	6.941	3	0.074
Affiliation[2]	0.555	0.171	3.250	11.954	1	0.001

[1] not indicated due to limited interpretability
[2] z-transformed to a mean of zero and a standard deviation of one
Significant effects ($p < 0.05$) are highlighted in bold.

5.5.2 Birds that passed control criterion

This model was not zero-inflated in contrast to the original model and thus we used a poisson distribution. Overdispersion was not issue (dispersion parameter: 0.08). The full model performed better than the null model (LRT: Chisq = 12.836, df = 5, p = 0.025).

Table S11. Summary of model results (including only birds that passed the criterion, N = 149 dyads) from the full model with number of prosocial acts as response variable and dyadic characteristics as predictors.

Term	Estimate	SE	Z	Chisq	df	p
Intercept	-5.887	0.905	-6.507			[1]
Species (BHM)	0.929	1.143	0.813			
speciesEC	2.216	1.197	1.851			
Species (GA)	-1.606	1.000	-1.606	20.301	3	<0.001
kinshipyes	-1.305	0.877	-1.488	2.406	1	0.121
sexFM	0.733	0.815	0.900			
sexMF	-0.882	0.824	-1.071			
sexMM	0.279	0.866	0.322	4.988	3	0.173
z.aff[2]	0.742	0.259	2.870	8.678	1	0.003

[1] not indicated due to limited interpretability
[2] z-transformed to a mean of zero and a standard deviation of one

Additional Discussion

Cheating behaviour

Individuals who repeatedly stepped on and off the provider perch were rewarded with food every sixth trial, and indeed, these "cheaters" acquired most of the food in the motivation trials. Consequently, the cheating individuals showed the same behaviour on every trial in both the test and the control conditions. However, this "cheating" strategy, would only lead to faster food acquisition in the control condition, as regular test trials would end if either two minutes had passed without a successful food delivery or if a group member acquired the food. One African grey parrot developed a different strategy to obtain more food rewards, but only during one session. This parrot repeatedly stole the sunflower seed from the beak of others during motivational trials. Our study is the first study to report this behaviour using the group service paradigm. Neither studies using the original version of the group service paradigm with primates (Burkart et al., 2014; Burkart & Van Schaik, 2013), nor studies using the adapted version of the group service paradigm with both corvids (Horn et al., 2016, 2020) and primates (Bhattacharjee et al., 2023; Martin et al., 2021) have reported this alternative 'cheating' strategy. A possible explanation for the observation of the alternative strategy in the present study could be the metal bar under the seesaw in the Galahs' and Eclectus' enclosure. Birds could remain in proximity to the provider perch by sitting next to it and could easily step on and off the perch. In the Horn et al. (2016, 2020) studies, but also in case of the two other parrot species that we have tested, there was no metal bar under the seesaw. Instead, another perch was installed on the receiver side for the birds to land on in order to get access to the food. Consequently, the birds had to fly to the provider perch and then fly off the perch again to start the next trial, which takes more energy than constantly stepping on and off the perch. Future studies using the group service paradigm with birds should be conducted without a surface to land on under the apparatus.

References

Bates, D., Maechler, M., Bolker, B., & Walker, S. (2014). lme4: Linear mixed-effects models using S4 classes. In *R package version 1.1-7*. http://cran.r-project.org/package=lme4

Bhattacharjee, D., Cousin, E., Pflüger, L. S., & Massen, J. J. M. (2023). Prosociality in a despotic society. *IScience*, *26*, 106587. https://doi.org/10.1016/j.isci.2023.106587

Brooks, M., Kristensen, K., van Benthem, K., Magnusson, A., Berg, C., Nielsen, A., Skaug, H., Maechler, M., & Bolker, B. (2017). glmmTMB Balances Speed and Flexibility Among Packages for Zero-inflated Generalized Linear Mixed Modeling. *The R Journal*, *9*(2), 378–400.

Burkart, J. M., Allon, O., Amici, F., Fichtel, C., Finkenwirth, C., Heschl, A., Huber, J., Isler, K., Kosonen, Z. K., Martins, E., Meulman, E. J., Richiger, R., Rueth, K., Spillmann, B., Wiesendanger, S., & Van Schaik, C. P. (2014). The evolutionary origin of human hyper-cooperation. *Nature Communications*, *5*, 1–9. https://doi.org/10.1038/ncomms5747

Burkart, J. M., & Van Schaik, C. (2013). Group service in macaques (Macaca fuscata), capuchins

(Cebus apella) and marmosets (Callithrix jacchus): A comparative approach to identifying proactive prosocial motivations. *Journal of Comparative Psychology*, *127*(2), 212–225. https://doi.org/10.1037/a0026392

Fox, J., & Weisberg, S. (2019). *An R Companion to Applied Regression* (3rd ed.). Thousand Oaks. https://socialsciences.mcmaster.ca/jfox/Books/Companion/

Holm, S. (1979). A Simple Sequentially Rejective Multiple Test Procedure. *Scandinavian Journal of Statistics*, *6*(2), 65–70.

Horn, L., Bugnyar, T., Griesser, M., Hengl, M., Izawa, E. I., Oortwijn, T., Rössler, C., Scheer, C., Schiestl, M., Suyama, M., Taylor, A. H., Vanhooland, L. C., von Bayern, A. M. P., Zürcher, Y., & Massen, J. J. M. (2020). Sex-specific effects of cooperative breeding and colonial nesting on prosociality in corvids. *ELife*, *9*, 235–244. https://doi.org/10.7554/eLife.58139

Horn, L., Scheer, C., Bugnyar, T., & Massen, J. J. M. (2016). Proactive prosociality in a cooperatively breeding corvid, the azure-winged magpie (*Cyanopica cyana*). *Biology Letters*, *12*(10), 20160649. https://doi.org/10.1098/rsbl.2016.0649

Martin, J. S., Koski, S. E., Bugnyar, T., Jaeggi, A. V., & Massen, J. J. M. (2021). Prosociality, social tolerance and partner choice facilitate mutually beneficial cooperation in common marmosets, Callithrix jacchus. *Animal Behaviour*, *173*, 115–136. https://doi.org/10.1016/j.anbehav.2020.12.016

Schielzeth, H., & Forstmeier, W. (2009). Conclusions beyond support: Overconfident estimates in mixed models. *Behavioral Ecology*, *20*(2), 416–420. https://doi.org/10.1093/beheco/arn145

Zeileis, A., Kleiber, C., & Simon, J. (2008). Regression Models for Count Data in R. *Journal of Statistical Software*, *27*(8).
